# An Electrochemical Determination of the Total Reducing Capacity of Wheat, Spelt, and Rye Breads

**DOI:** 10.3390/antiox11081438

**Published:** 2022-07-25

**Authors:** Danuta Zielińska, Henryk Zieliński, Mariusz Konrad Piskuła

**Affiliations:** 1 Department of Chemistry, University of Warmia and Mazury in Olsztyn, Plac Łódzki 4, 10-721 Olsztyn, Poland; danuta.zielinska@uwm.edu.pl; 2 Department of Chemistry and Biodynamics of Food, Division of Food Sciences, Institute of Animal Reproduction and Food Research, Polish Academy of Sciences, Tuwima 10, 10-748 Olsztyn, Poland; h.zielinski@pan.olsztyn.pl

**Keywords:** breads, crumbs, crusts, cyclic voltammetry, total reducing capacity

## Abstract

The most interesting activities associated with bread components such as phenolic compounds, fibre, tocols, or newly formed compounds in the Maillard reaction, are their reducing properties responsible for the formation of the overall reducing capacity of bread. Among the electrochemical methods, the cyclic voltammetry (CV) technique has been recently adapted for this purpose. In this study, the application of the CV assay for the determination of the total reducing capacity of flours, doughs, and breads as well as their crumbs and crusts, originated from wheat, spelt, and rye formulated on white flours (extraction rate of 70%) and dark flours (extraction rate of 100%) and baked at 200 °C for 35 min and at 240 °C for 30 min was addressed. The reducing capacity of hydrophilic extracts from white flours and breads as well as their crumbs and crusts showed double values when compared to that of lipophilic ones whilst hydrophilic and lipophilic extracts from dark breads and their parts revealed comparable levels. The dark wheat, spelt, and rye breads showed an approximately threefold higher total reducing capacity than white breads. Baking at higher temperature slightly increased the total reducing capacity of breads and the highest value was found for dark rye bread as well as its crust baked at 240 °C for 30 min. The cyclic voltammetry methodology showed to be especially suitable for screening the bread technology and allows for obtaining rapid electrochemical profiles of bread samples.

## 1. Introduction

Cereals form the basis of the nutrition pyramid. They are the most important and staple food for the majority of mankind. They are sources of carbohydrates, proteins, lipids rich in essential fatty acids, vitamins, especially many B vitamins, minerals, fiber, antioxidants, and phytochemicals. Wheat (*Triticum aestivum*) is by far the most important crop for breadmaking; however, spelt wheat (*Triticum aestivum* ssp. *spelta*) is also of increasing concern [1]. The nutritive value of bread could be increased if, for example, rye (*Secale cereale*) is used for breadmaking [2,3].

At present, the antioxidant properties of food samples can be determined by several methods [4,5,6,7,8]. Among others, scavenging capacity assays against stable, non-biological radicals such as DPPH**^•^** and ABTS^•+^, scavenging capacity assays against specific reactive oxygen species such as the superoxide radical anion scavenging capacity, the hydrogen peroxide scavenging capacity, the hydroxyl radical scavenging capacity, the hypochlorous acid scavenging capacity, the singlet oxygen scavenging capacity, the total radical trapping antioxidant parameter (TRAP), the ferric reducing antioxidant power (FRAP), and the cupric ions (Cu^2+^) reducing power (CUPRAC) were the most employed in foods [6]. Currently, the methods used for determination of the reducing capacity of food samples are based on the application of the Folin–Ciocalteu phenol reagent (spectrophotometric FC assay) and electrochemical techniques [6,9,10,11,12,13,14].

Among different electrochemical techniques such as cyclic voltammetry (CV), differential pulse voltammetry (DPV), and square wave voltammetry (SWV), the CV has been adapted to the evaluation of the overall reducing capacity of food extracts and other biological samples [9,15].

The reducing capacity estimated by electrochemical methods represents the overall capability of the sample to donate electrons (oxidation potential) as well as the overall concentration of the reducing species responsible for this ability (anodic current). Usually, voltametric data (amperometric and coulometric) provides two parameters. The first is the peak or half-wave potential of anodic oxidation and the second is the current limit or peak current, which reflects the concentration of antioxidants. The potentials mentioned are directly related to the potential of the formal redox system, *E*^0′^. They make it possible to assess whether the oxidation reactions occurs. The formal potential can thus be regarded as a measure of antioxidant power [16]. 

The total reducing capacity (TRC) of the sample is a function of two parameters derived from CV voltammograms. The first is the oxidation potential (*E*_p,a_) and the second is the intensity of the anodic AC current (*I*_a_). Anodic current (*I*_a_) is directly proportional, at any potential, to the concentration of the compound in the solution and analytically it is used to determine its concentration. However, more often the area under the AC wave (S; related to the total charge) is used since it is a more relevant parameter responding to the reducing capacity of the sample [15].

The CV assay allows rapid screening of the electrochemical profile and is especially suitable for screening studies on biological samples. The importance/advantages of the application of this technique for the evaluation of the total reducing capacity of a food sample based on CV profile obtained both in aqueous medium and in organic solvents provided that there are redox-active components and enough electrolytes in the solution to support redox reactions on the electrode surface [6]. The novelty of this manuscript is based on the application of the CV technique for the determination of the total reducing capacity (TRC), calculated as the sum of reducing capacity obtained for hydrophilic (H-RC) and lipophilic (L-RC) food extracts.

Therefore, the aim of the study was to show the application of the CV assay for the determination of the total reducing capacity (TRC) of wheat, spelt, and rye flours and breads as well as their crumbs and crusts, formulated on white flours (extraction rate of 70%) and dark flours (extraction rate of 100%) and baked at 200 °C for 35 min and at 240 °C for 30 min. 

## 2. Materials and Methods

### 2.1. Chemicals

HPLC-grade methanol, n-hexane, acetic acid (supra-gradient), and sodium acetate were from Merck KGaA (Darmstadt, Germany). Water was purified with an ili-Q-system (Milipore, Bedford, MA, USA). Trolox (6-hydroxy-2,5,7,8-tetramethylchroman-2-carboxylic acid) was purchased from Sigma (Sigma Chemical Co., St. Louis, MO, USA). All other reagents were obtained from POCh, (Gliwice, Poland). 

### 2.2. Rye, Wheat, and Spelt Breads Baking Methods

White wheat, spelt, and rye breads were formulated on corresponding flours with an extraction rate of 70%, whereas dark wheat, spelt, and rye breads on flours with an extraction rate of 100%. The methods for dough preparation and type of fermentation used for above breads preparations were described in detail by Przygodzka et al. [17]. Breads were baked in an electric oven (DC-32E, Sveba Dahlen, Sweden) at 200 °C for 35 min or at 240 °C for 30 min. Breads were cut into slices about 1 cm thick and then from the top central surface the crumb and crust were separated. The slices, crumbs, and crusts were freeze-dried, powdered, and finally stored at −20 °C until analysis for no longer than four weeks.

### 2.3. Sample Preparation for Measurement of the Total Reducing Capacity by Cyclic Voltammetry 

Exactly 250 mg of dried and pulverized sample was extracted at room temperature with 1 mL of aqueous methanol (1:4; *v/v*) or 1 mL of mixture n-hexane and methanol (1:4; *v/v*) to obtain hydrophilic or lipophilic extracts, respectively. The sonication for 60 s was applied. The Sonic Vibra Cell (model VC 750, Qsonica L.L.C**,** Newtown, CT, USA) set on 100% amplitude corresponded to high frequency vibration of 20 kHz. Then, mixtures were vortexed for 60 s, again sonicated, and finally centrifuged for 5 min (5000× *g*, 4 °C). This step was repeated five times on the residue with the next volume of 1.0 mL of the proper solvent. Supernatants were collected in a 5-mL flask. 

### 2.4. Cyclic Voltammetry Assay

Electrochemical measurements were performed using the G 750 potentiostat/galvanostat (Gamry Ins., Warminster, PA, USA). A conventional system composed of three electrodes was used for the cyclic voltametric assay. A glassy carbon (GC) as a working electrode (BAS MF-2012), a saturated Ag/AgCl electrode as the reference, and a Pt wire as the counter electrode. Before each experiment, the GC electrode was polished with 0.05 μm alumina paste (Polishing alumina, BAS) and then ultrasonically rinsed in deionized water. The electrochemical measurements were carried out in solutions prepared by mixing the 100 μL of analyzed extracts with the same volume of 0.2 M sodium acetate–acetic buffer (pH 4.5). Simultaneously, buffer acts as a supporting electrolyte. 

**Figure 1:**
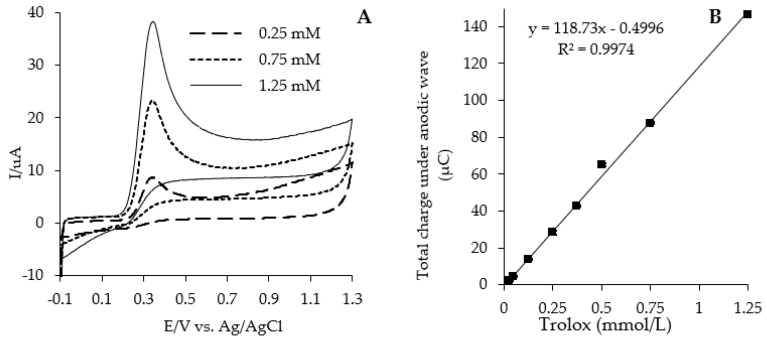
(**A**) Cyclic voltammograms of Trolox concentration (0.25–1.25 mM) in aqueous methanol (1:4, *v/v*) and 0.1 M sodium acetate–acetic buffer (pH 4.5); scan rate 100 mV∙s^−1^. (**B**) The dependency of the total charge under the anodic wave (area below anodic wave) as a function of increasing concentration of Trolox.

Cyclic voltammograms were recorded by scanning the potential in the range from −100 to +1200 mV at a scan rate of 100 mV s^−1^. For the needs of the CV assay, the peak area below the anodic wave of the voltammogram was calculated within the range of +100 to +1100 mV. The aqueous methanol (1:4, *v/v*) and hexane/methanol (4:1, *v/v*) Trolox solutions within the concentration range of 0.10–1.25 mM were used to develop the respective standard curves (Figure 1A,B). The reducing capacities were estimated in terms of the concentration of Trolox solution producing the same peak area than the oxidation peak recorded in 100 mg/mL solutions of the extracts.

TRC was calculated as the sum of reducing capacity values obtained for hydrophilic (H-RC) and lipophilic (L-RC) extracts of bread samples, and it was expressed in terms of μmol Trolox/g dry matter (DM).

### 2.5. Statistical Analysis

Results are displayed as the mean ± standard deviation of three independent measurements. Statistical analyses were performed by one-way analysis ANOVA with Fischer LSD test with the significance level set at *p* < 0.05. The Statistica 7.1.30.0 software (StatSoft Inc., Tulsa, OK, USA, 2001) for Windows was used. 

## 3. Results and Discussion

In this study, the CV method was applied for the determination of the total reducing capacity of flours, doughs, and breads as well as their crumbs and crusts, originated from wheat, spelt, and rye formulated on white flours (extraction rate of 70%) and dark flours (extraction rate of 100%) and baked at 200 °C for 35 min and at 240 °C for 30 min. The target compounds for electrochemical methods are phenolic compounds mainly present in a common redox behavior, for which electrochemical oxidation occurs at the –OH groups and is influenced by the chemical substituents linked to the aromatic rings (e.g., –OCH_3_, sugar, etc.). The phenolic –OH moiety undergoes anodic oxidation according to the stability of the electrogenerated phenoxy radical, which is dependent on phenol and phenolic substituents [18].

### 3.1. The Overall Reducing Capacity of White and Dark Flours and Breads Provided by Cyclic Voltammetry

**Figure 2:**
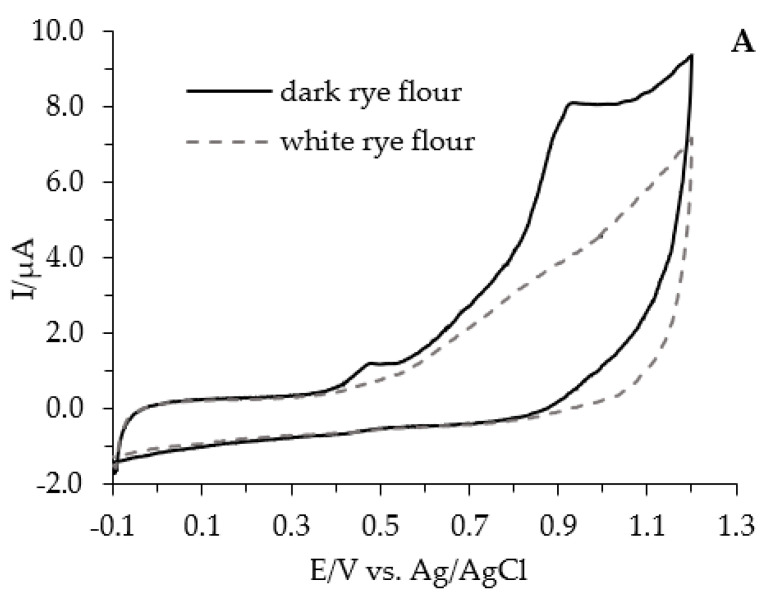

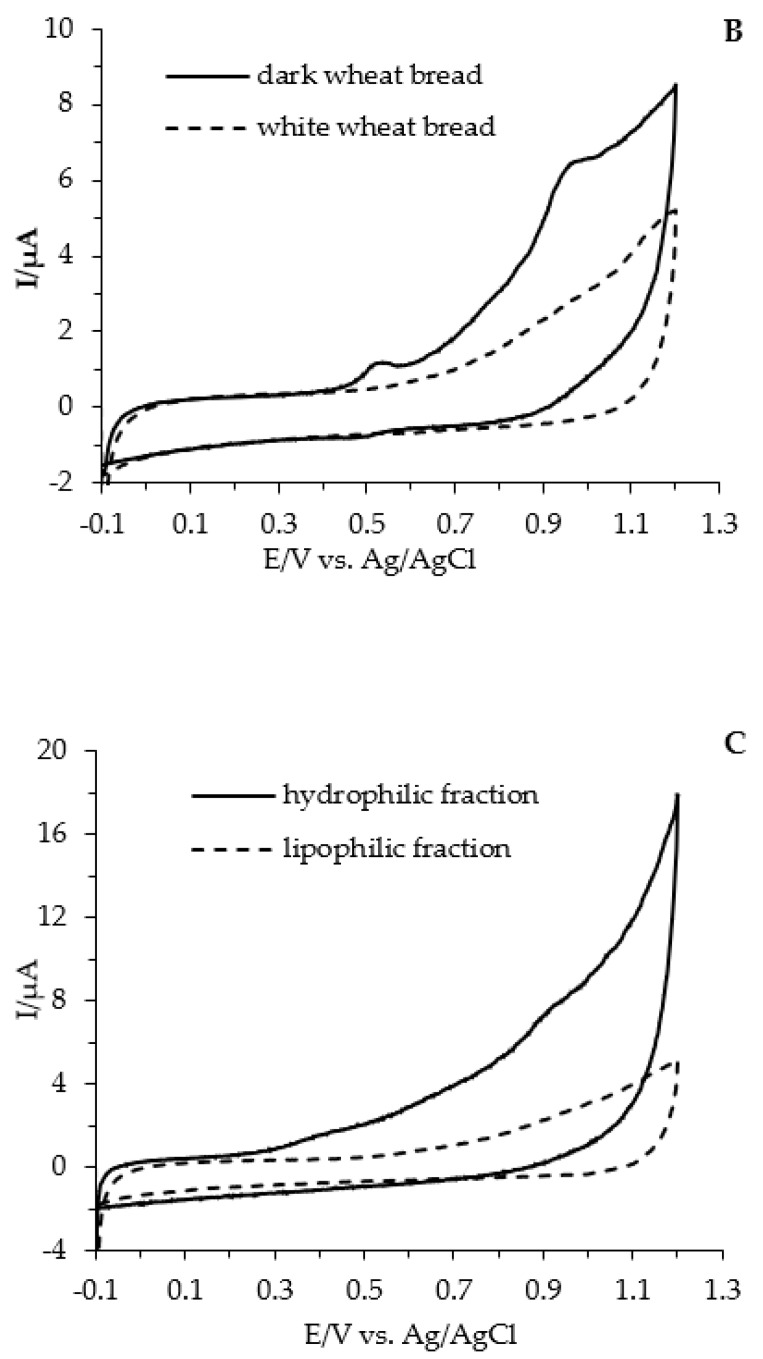
Cyclic voltammograms of hydrophilic extracts originated from white and dark rye flour (**A**), cyclic voltammograms of hydrophilic extracts of slices originated from white and dark wheat bread (200 °C/35 min.) (**B**), cyclic voltammograms of the hydrophilic and lipophilic fraction of crust separated from white wheat bread (240 °C/35 min.) (**C**). Concentration of extract: 100 mg/mL; sample preparation: hydrophilic fraction: aqueous methanol (1:4, *v/v*) extract mixed with 0.2 M acetic buffer (pH 4.5) at ratio 1:1 (*v/v*); lipophilic fraction: methanol/hexane 4:1 (*v/v*) extract mixed with 0.2 M acetic buffer (pH 4.5) at ratio 1:1 (*v/v*); and scan rate 100 mV/s.

The total reducing capacity of white and dark flours and breads as well as their slices, crumbs, and crusts was formed by hydrophilic and lipophilic reducing species. For example, the cyclic voltammograms of hydrophilic extracts originated from white and dark rye flour (A), cyclic voltammograms of hydrophilic extracts of slices originated from white and dark wheat bread (200 °C/35 min.) (B), and cyclic voltammograms of the hydrophilic and lipophilic fraction of crusts separated from white wheat bread (240 °C/35 min.) (C) are shown on Figure 2.

In the same manner were provided cyclic voltammograms of hydrophilic and lipophilic extracts of slices, crumbs, and crusts originating from wheat, spelt, and rye breads baked at 200 °C for 35 min or at 240 °C for 30 min. 

The observed CV voltammograms were irreversible and anodic waves were broadened because of the response of several antioxidants with different oxidation potentials. According to our previously published papers as well as by other authors, it is known that wheat, spelt, and rye flour contain potentially beneficial antioxidants, including tocopherols and tocotrienols, phenolic acids, and in lower extend flavonoids, reduced glutathione, and inositol hexaphosphates [2,3]. There are three technological steps, namely dough preparation, dough fermentation, and baking, which may have an impact on the profile and content of antioxidants/reductants compounds. The flour and respective breads show almost the same profile of phenolic acids, including caffeic, ferulic, p-coumaric, sinapic, and vanillic acids [2,3]. In contrast, the loss of the content of tocopherols and tocotrienols was noted and this effect could result from their reduction during dough fermentation with air contact and thermal treatment during baking [19,20]. Moreover, the losses of phytic acid during bread preparation was observed, which was connected with the action of phytase [19]. The level of glutathione in flours was low, it still plays a significant role in redox reactions occurring in flour as well as in bread making [21]. It was proven that that bread making affects the antioxidant capacity of breads due to Maillard reaction products (MRPs) formation mainly concerned with the bread crust. Advanced MRPs resulted also as a good scavenger of peroxyl and ABTS radicals, whereas early MRPs seem to be not connected to the antioxidant activity. Advanced MRPs resulted as also a good scavenger of peroxyl and ABTS radicals whereas early MRPs seem to be not connected to the antioxidant activity [22]. 

### 3.2. The Total Reducing Capacity (TRC) Values of White and Dark Flours and Breads Based on the Cyclic Voltammetry

In this study, having a completed cyclic voltammograms, the TRC was calculated as the sum of reducing capacity values obtained for H-RC and L-RC extracts of flours and bread samples, and it was expressed in terms of μmol Trolox/g DM. For this purpose, a respective aqueous methanol (1:4, *v/v*) and hexane/methanol (4:1, *v/v*) Trolox solutions within the concentration range of 0.10–2.5 mM were used to develop and apply the respective standard curves as it is shown in Figure 1.

The reducing capacity of hydrophilic (H-RC) and lipophilic (L-RC) fractions from flours, slices, crumbs, and crusts originated from white and dark wheat, spelt, and rye breads baked at 200 °C for 35 min or at 240 °C for 30 min (μmol Trolox/g DM) are shown in Table 1 and Table 2, whereas TRC data are presented in Table 3.

**Figure 3:**
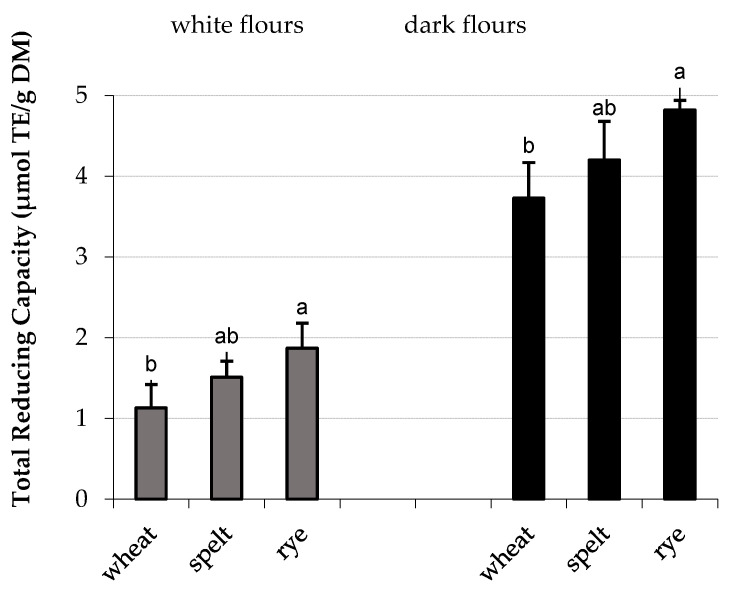
The total reducing capacity of white and dark flours originated from wheat, spelt, and rye. Data are the mean of three independent replicates ± SD. Similar letter as superscripts for bars related to the white or dark flours indicates no significant differences (*p* ≤ 0.05).

The study showed that the TRC of flours was formed both by electroactive components of hydrophilic (H-RC) and lipophilic (L-RC) extracts. Among white flours of an extraction rate of 70%, the H-RC fraction from wheat and spelt flours was a richer source of electroactive substances as compared to the L-RC fractions. In contrast, the L-RC fractions of wheat, spelt, and rye flours of an extraction rate of 100% were the richest source of electroactive components. Based on the cyclic voltammograms of hydrophilic and lipophilic extracts, it was shown that dark flours showed an almost threefold higher total reducing capacity than white flours. Among the dark flours, the higher total reducing capacity showed the dark rye flour (Figure 3). The order of the total reducing capacities of flours was as follows: rye flour > spelt flour > wheat flour.

**Figure 4:**
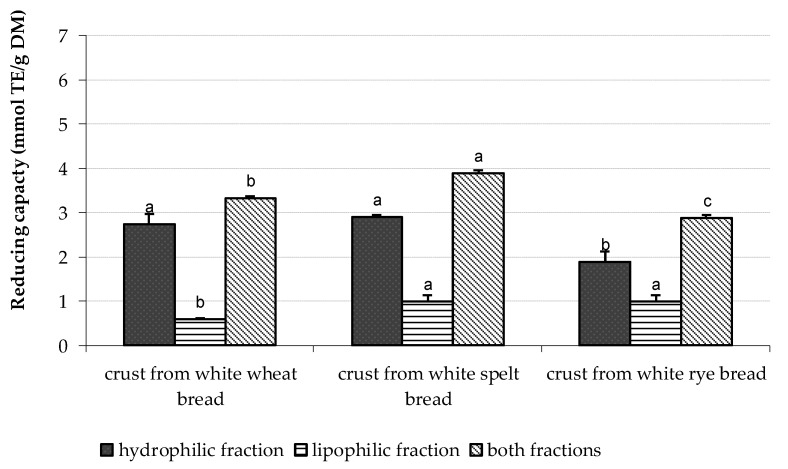
The total reducing capacity of crusts separated from white breads baked at 240 °C for 30 min. Data are the mean of three independent replicates ± SD. Similar letter as superscripts for bars related to the reducing capacity of hydrophilic or lipophilic or TRC indicates no significant differences (*p* ≤ 0.05).

**Figure 5:**
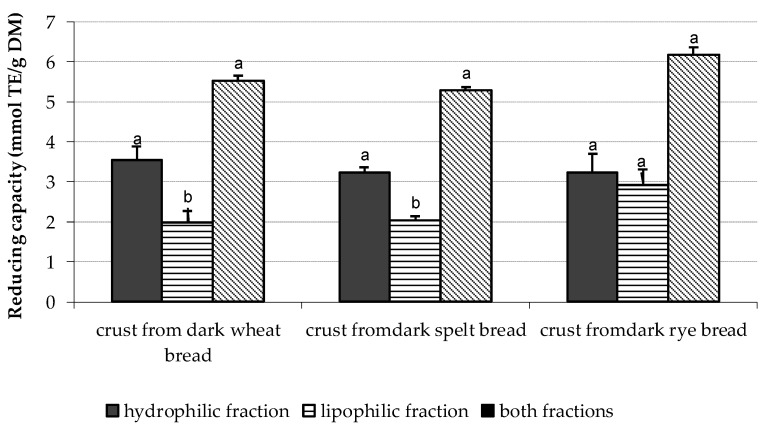
The total reducing capacity of crusts separated from dark breads baked at 240 °C for 30 min. Data are the mean of three independent replicates ± SD. Similar letter as superscripts for bars related to the reducing capacity of hydrophilic or lipophilic or TRC indicates no significant differences (*p* ≤ 0.05).

The study showed that the TRC of dough prepared from white flours increased by 34–66%, whereas those formed from dark flours increased by 15–32% and the noted increases were the type flour-dependent. The next observations were made to the baking process. The obtained breads showed a higher TRC as compared to the respective flours. The noted increased TRC values were dependent on the type of bread (white vs dark), the time of baking, and the temperature used. It was observed that white rye bread baked at a lower temperature at 200 °C for 35 min and dark rye bread baked at 240 °C for 30 min showed the highest TRC values. No impact of baking time and temperature was observed in relation to wheat and spelt breads prepared from white and dark flours. The TRC of dark rye bread baked at 240 °C for 30 min was the highest among all bread analyzed. It is worthy to note that the TRC of dark breads prepared from wheat, spelt, and rye was threefold higher as compared to the respective white breads. Przygodzka et al. [17], searching for the factors influencing acrylamide formation in breads, showed the positive correlation between the total phenolic compounds (TPC) and the antioxidant capacity of wheat, spelt, and rye breads provided by the ABTS assay and the photochemiluminescence assay. The content of TPC was also related to the calculated TRC of breads. As the TPC of breads was higher, then also the TRC was increased. Regarding the parts of bread, the following TRC rank was noted: crust > crumb> slice. This rank was with agreement to our published data on TRC provided by other total antioxidant capacity assays, different than CV [2]. Moreover, our data clearly indicate that the TRC of crusts was mainly formed by an electroactive substance present in the hydrophilic (H-RC) fraction as it is presented on Figure 4 and Figure 5.

The study showed that the CV technique can be widely used for the measurement of the total reducing capacity of bread samples as well as their separated parts such as crumb and crust. The main advantage of the CV technique is its capability to rapidly observe the total redox behavior over a wide potential range without the necessity of measuring the specific reducing activity of each component alone. Moreover, the CV assay is low-cost and usually does not require time consuming sample preparation. However, it should be mentioned that practical limitation of applied CV methodology was that the working electrode had to be frequently cleaned to remove residues of sample from its surface and to maintain its sensitivity.

Previously, we showed the application of the cyclic voltammetry (CV) technique for the determination of the reducing capacity of buckwheat-enhanced gluten-free bread, gluten-free muffins, and the bioaccessible reducing capacity of buck-wheat-enhanced white and dark wheat breads [23,24,25,26,27]. The CV technique was also applicable for the determination of changes in the reducing capacity of the processed food samples such as raw and roasted buckwheat groats [26], high pressure-assisted enzymatic released peptides of pinto bean hydrolysates [27], as well as the description of antioxidant properties of caffeic acid [28]. All this evidence strongly supports our view and other authors on the useful CV technique in food science and technology [18].

## 4. Conclusions

We showed that the CV methodology was suitable for obtaining a rapid electrochemical profile of a bread sample as well as its parts for evaluating their TRC. Therefore, the TRC of breads and their parts seems to be an important factor characterizing the functional properties of bread among others. The TRC was a useful chemical marker for screening the bread technology and to conclude whether baking at a shorter time and at a higher temperature was better than baking for a longer time at a lower temperature. This paper also strongly supported our view as well as other authors on the useful CV technique in food science and technology.

## Figures and Tables

**Table 1:** The reducing capacity of hydrophilic fraction (H-RC) from flours, doughs, slices, crumbs, and crusts originated from white and dark wheat, spelt and rye breads baked at 200 °C for 35 min or at 240 °C for 30 min (μmol Trolox/g DM).

Type of Bread	Baking Conditions	Flour	Dough	Bread Slice	Bread Crumb	Bread Crust
White wheat bread	200 °C/35 min 240 °C/30 min	0.75 ± 0.15 ^A^	1.16 ± 0.13 ^A^	1.44 ± 0.06 ^b,A^ 1.18 ± 0.12 ^a,A^	1.13 ± 0.07 ^a,A^ 1.15 ± 0.13 ^a,A^	1.40 ± 0.17 ^a,A^ 2.74 ± 0.23 ^b,A^
Dark wheat bread	200 °C/35 min 240 °C/30 min	1.68 ± 0.30 ^B^	2.32 ± 0.09 ^B^	2.36 ± 0.02 ^a,B^ 2.44 ± 0.11 ^a,B^	2.44 ± 0.19 ^a,B^ 2.30 ± 0.01 ^a,B^	4.17 ± 0.26 ^a,B^ 3.54 ± 0.34 ^a,B^
White spelt bread	200 °C/35 min 240 °C/30 min	0.83 ± 0.06 ^A^	1.01 ± 0.25 ^A^	1.04 ± 0.12 ^a,A^ 1.15 ± 0.08 ^a,A^	1.14 ± 0.17 ^a,A^ 1.04 ± 0.18 ^a,A^	1.57 ± 0.09 ^a,A^ 2.90 ± 0.04 ^b,A^
Dark spelt bread	200 °C/35 min 240 °C/30 min	1.94 ± 0.23 ^B^	1.99 ± 0.28 ^B^	2.37 ± 0.34 ^a,B^ 2.10 ± 0.06 ^a,B^	2.26 ± 0.02 ^b,B^ 2.03 ± 0.09 ^a,B^	2.81 ± 0.16 ^a,B^ 3.24 ± 0.29 ^a,B^
White rye bread	200 °C/35 min 240 °C/30 min	0.89 ± 0.07 ^A^	1.25 ± 0.15 ^A^	1.51 ± 0.03 ^b,A^ 1.28 ± 0.01 ^a,A^	1.55 ± 0.06 ^b,A^ 1.33 ± 0.01 ^a,A^	1.71 ± 0.01 ^a,A^ 1.89 ± 0.04 ^b,A^
Dark rye bread	200 °C/35 min 240 °C/30 min	2.16 ± 0.09 ^B^	2.87 ± 0.02 ^B^	2.66 ± 0.10 ^a,B^ 2.99 ± 0.19 ^a,B^	3.04 ± 0.17 ^b,B^ 2.64 ± 0.02 ^a,B^	3.35 ± 0.11 ^a,B^ 3.24 ± 0.13 ^a,B^

Values are means of three determinations ± standard deviation. Values in each column of indicated type of bread with the same small superscript are not different (*p* > 0.05). Values in each column with the same capital superscript for respective white and dark wheat, spelt, and rye breads baked in the same temperature (200 °C or 240 °C) are not different (*p* > 0.05).

**Table 2:** The reducing capacity of the lipophilic (L-RC) fraction from flours, doughs, slices, crumbs, and crusts originated from white and dark wheat, spelt, and rye breads baked at 200 °C for 35 min or at 240 °C for 30 min (μmol Trolox/g DMr).

Type of Bread	Baking Conditions	Flour	Dough	Bread Slice	Bread Crumb	Bread Crust
White wheat bread	200 °C/35 min 240 °C/30 min	0.38 ± 0.14 ^A^	0.73 ± 0.06 ^A^	0.52 ± 0.05 ^a,A^ 0.63 ± 0.04 ^b,A^	0.72 ± 0.05 ^b,A^ 0.50 ± 0.03 ^a,A^	0.87 ± 0.13 ^b,A^ 0.59 ± 0.06 ^a,A^
Dark wheat bread	200 °C/35 min 240 °C/30 min	2.05 ± 0.19 ^B^	2.52 ± 0.14 ^B^	2.02 ± 0.11 ^a,B^ 1.96 ± 0.10 ^a,B^	2.21 ± 0.09 ^a,B^ 2.17 ± 0.12 ^a,B^	1.85 ± 0.08 ^a,B^ 1.99 ± 0.12 ^a,B^
White spelt bread	200 °C/35 min 240 °C/30 min	0.68 ± 0.13 ^A^	1.02 ± 0.02 ^A^	0.80 ± 0.02 ^a,A^ 0.83 ± 0.03 ^a,A^	0.87 ± 0.07 ^a,A^ 0.86 ± 0.09 ^a,A^	0.78 ± 0.01 ^a,A^ 0.99 ± 0.15 ^a,A^
Dark spelt bread	200 °C/35 min 240 °C/30 min	2.25 ± 0.28 ^B^	2.84 ± 0.05 ^B^	2.39 ± 0.07 ^a,B^ 2.37 ± 0.20 ^a,B^	2.29 ± 0.07 ^a,B^ 2.32 ± 0.09 ^a,B^	1.85 ± 0.11 ^a,B^ 2.04 ± 0.10 ^a,B^
White rye bread	200 °C/35 min 240 °C/30 min	0.98 ± 0.07 ^A^	1.36 ± 0.11 ^A^	1.31 ± 0.01 ^b,A^ 0.98 ± 0.02 ^a,A^	1.19 ± 0.06 ^b,A^ 1.10 ± 0.01 ^a,A^	0.97 ± 0.05 ^a,A^ 0.99 ± 0.07 ^a,A^
Dark rye bread	200 °C/35 min 240 °C/30 min	2.66 ± 0.15 ^B^	3.51 ± 0.06 ^B^	3.07 ± 0.01 ^a,B^ 2.95 ± 0.05 ^a,B^	3.13 ± 0.11 ^a,B^ 3.05 ± 0.05 ^a,B^	2.88 ± 0.11 ^a,B^ 2.93 ± 0.09 ^a,B^

Values are means of three determinations ± standard deviation. Values in each column of indicated type of bread with the same small superscript are not different (*p* > 0.05). Values in each column with the same capital superscript for respective white and dark wheat, spelt, and rye breads baked in the same temperature (200 °C or 240 °C) are not different (*p* > 0.05).

**Table 3:** The total reducing capacity (TRC) of flours, doughs, slices, crumbs, and crusts originated from white and dark wheat, spelt, and rye breads (μmol Trolox/g DM).

Type of Bread	Baking Conditions	Fluor	Dough	Bread Slice	Bread Crumb	Bread Crust
White wheat bread	200 °C/35 min 240 °C/30 min	1.13 ± 0.29 ^A^	1.88 ± 0.19 ^A^	1.96 ± 0.11 ^a^^,^^A^ 1.81 ± 0.16 ^a,A^	1.85 ± 0.12 ^a,A^ 1.65 ± 0.16 ^a,A^	2.27 ± 0.30 ^a,A^ 3.33 ± 0.29 ^b,B^
Dark wheat bread	200 °C/35 min 240 °C/30 min	3.73 ± 0.49 ^B^	4.84 ± 0.23 ^B^	4.37 ± 0.13 ^a,A^ 4.41 ± 0.21 ^a,A^	4.65 ± 0.28 ^a,A^ 4.47 ± 0.13 ^a,A^	6.02 ± 0.34 ^b,A^ 5.53 ± 0.46 ^a,A^
White spelt bread	200 °C/35 min 240 °C/30 min	1.51 ± 0.19 ^A^	2.03 ± 0.38 ^A^	1.83 ± 0.14 ^a,A^ 1.98 ± 0.11 ^a,A^	2.01 ± 0.24 ^a,A^ 1.91 ± 0.27 ^a,A^	2.36 ± 0.10 ^b,A^ 3.89 ± 0.19 ^b,B^
Dark spelt bread	200 °C/35 min 240 °C/30 min	4.20 ± 0.51 ^B^	4.84 ± 0.33 ^B^	4.76 ± 0.38 ^a,A^ 4.47 ± 0.26 ^a,A^	4.56 ± 0.09 ^a,B^ 4.35 ± 0.18 ^a,A^	4.66 ± 0.27 ^a,A^ 5.27 ± 0.39 ^b,B^
White rye bread	200 °C/35 min 240 °C/30 min	1.87 ± 0.14 ^A^	2.62 ± 0.26 ^A^	2.82 ± 0.04 ^a,A^ 2.26 ± 0.03 ^a,B^	2.74 ± 0.07 ^a,B^ 2.43 ± 0.02 ^a,A^	2.69 ± 0.06 ^a,A^ 2.88 ± 0.11 ^a,A^
Dark rye bread	200 °C/35 min 240 °C/30 min	4.83 ± 0.24 ^B^	6.39 ± 0.08 ^B^	5.72 ± 0.11 ^a,A^ 5.94 ± 0.24 ^a,A^	6.17 ± 0.28 ^b,B^ 5.69 ± 0.07 ^a,A^	6.23 ± 0.22 ^b,A^ 6.17 ± 0.22 ^a,A^

Values are means of three determinations ± standard deviation. Values in each column of indicated type of bread with the same small superscript are not different (*p* > 0.05). Values in each column with the same capital superscript for respective white and dark wheat, spelt, and rye breads baked in the same temperature (200 °C or 240 °C) are not different (*p* > 0.05).

## Data Availability

The data presented in this study are available in the article.
